# A Curated, Comprehensive Database of Plasmid Sequences

**DOI:** 10.1128/MRA.01325-18

**Published:** 2019-01-03

**Authors:** Lauren Brooks, Mo Kaze, Mark Sistrom

**Affiliations:** a University of California Merced, Merced, California, USA; b Utah Valley University, Orem, Utah, USA; University of Maryland School of Medicine

## Abstract

Plasmid sequences are central to a myriad of microbial functions and processes. Here, we have compiled a database of complete plasmid sequences and associated metadata curated from both NCBI’s recent genome database update, which includes plasmids as organisms, and all available annotated bacterial genomes. The resultant database contains 10,892 complete plasmid sequences and associated metadata.

## Abstract (precis)

Plasmid sequences are central to a myriad of microbial functions and processes. Here, we have compiled a database of complete plasmid sequences and associated metadata curated from both NCBI’s recent genome database update, which includes plasmids as organisms, and all available annotated bacterial genomes.

## ANNOUNCEMENT

Plasmids are one of the key vectors of horizontal gene transfer in bacteria and archaea (1). Plasmids play a major role in bacterial genetic diversity (2), evolution (3), and adaptation (4). Conjugative exchange (i.e., the transfer of plasmids from one bacterium to another) can lead to the spread of a variety of functions, including degradation of heavy metals and anthropogenic toxic waste (5), bacteriocin and toxin production to ward off predators (6), and, alarmingly, antibiotic resistance and virulence plasmids that inhibit antibiotics and lead to novel and untreatable diseases (7). Plasmids are also extensively used as tools in genetic engineering (8).

To generate a comprehensive plasmid database, we started with the recent NCBI genome database update, which has a separate collection of plasmids as organisms. FASTA format files containing plasmid “genome” sequences were downloaded on 5 March 2018 from ftp://ftp.ncbi.nlm.nih.gov/refseq/release/plasmid/, resulting in 11,677 plasmid sequences. Using the R package Rentrez (https://cran.r-project.org/web/packages/rentrez/index.html), we downloaded the metadata available from the nucleotide database for each entry based on the locus number contained in the header file for each plasmid. Metadata from the BioProject, BioSample, and Assembly databases were also pulled for each plasmid sequence when present. An initial review of the metadata demonstrated that not all sequences contained in the downloaded files were complete plasmid sequences. After downloading all sequences labeled as plasmids (*n* = 11,677), we filtered the database using the nucleotide metadata to remove partial plasmid sequences from the databases (*n* = 9,763) and again using the assembly metadata to remove incomplete assemblies (*n* = 7,434). Additionally, 8 sequences labeled as phages were found and removed from the database. This resulted in 7,426 complete and assembled plasmid sequences following this initial screening.

In addition to curating the predefined NCBI plasmid database, we extracted plasmid sequences from bacterial genomes with complete assemblies in NCBI’s prokaryotic genome database (https://www.ncbi.nlm.nih.gov/genome/browse#!/prokaryotes/). Genomic assemblies labeled as partially complete or in contigs were not included to ensure that only complete plasmid sequences were included in our final database. Sequences that were already included as part of the original plasmid downloads, as identified by their accession or locus numbers, were removed as duplicates. This allowed us to include an additional 3,466 complete, annotated plasmid sequences, resulting in our database of 10,892 complete and annotated plasmid sequences for subsequent analyses.

The two data sets described above were combined to result in a comprehensive, complete, and annotated plasmid database. Metadata for this final list were compiled using the accession version number provided in the header for each plasmid sequence as described above.

### Data availability.

The plasmid database is available in fasta format and associated metadata are available in csv format at https://doi.org/10.15146/R33X2J.
